# Sex Differences in Immunity to Viral Infections

**DOI:** 10.3389/fimmu.2021.720952

**Published:** 2021-08-31

**Authors:** Henning Jacobsen, Sabra L. Klein

**Affiliations:** W. Harry Feinstone Department of Molecular Microbiology and Immunology, The Johns Hopkins Bloomberg School of Public Health, Baltimore, MD, United States

**Keywords:** inflammation, innate immunity, T cells, antibodies, SARS-CoV-2, influenza

## Abstract

The ongoing COVID-19 pandemic has increased awareness about sex-specific differences in immunity and outcomes following SARS-CoV-2 infection. Strong evidence of a male bias in COVID-19 disease severity is hypothesized to be mediated by sex differential immune responses against SARS-CoV-2. This hypothesis is based on data from other viral infections, including influenza viruses, HIV, hepatitis viruses, and others that have demonstrated sex-specific immunity to viral infections. Although males are more susceptible to most viral infections, females possess immunological features that render them more vulnerable to distinct immune-related disease outcomes. Both sex chromosome complement and related genes as well as sex steroids play important roles in mediating the development of sex differences in immunity to viral infections.

## Introduction

The ongoing coronavirus infectious disease 2019 (COVID-19) pandemic has raised awareness about sex-specific differences in immunity against severe acute respiratory syndrome coronavirus 2 (SARS-CoV-2) infection. Although individual countries report sex biases in rates of confirmed infections (1) on a global scale, infection rates appear to be similar between males and females (2). A more consistent observation across diverse countries and cultures is that severe COVID-19 is more likely to occur in males than females (3–8). Male COVID-19 patients are twice as likely to require ICU admission and are 30% more likely to die due to COVID-19 compared to female patients (9). Sex differences in outcomes of viral infections are not limited to SARS-CoV-2 and numerous clinical and epidemiological studies have provided evidence that biological sex broadly affects immunity to viral infection (10–12). Infections with viruses, such as Dengue virus, hantaviruses, and hepatitis B (HBV) and C (HCV) viruses, are more prevalent in human males than females suggesting that behavioral or occupational exposures contribute (13–17). The intensity and severity of disease caused by some viruses, including but not limited to Epstein Barr virus, HBV, HCV, and West Nile virus, are also greater for males than females (16–21). There are, however, some viruses that are more prevalent in females than males, such as cytomegalovirus, herpes simplex virus type 2 (HSV2), and human T-cell leukemia virus type 1 (22–25), and that cause more severe disease following infection, including hantaviruses, HSV2, human immunodeficiency virus (HIV), pandemic influenza A viruses (IAVs), and measles virus (14, 23, 24, 26–29). With regard to beta coronaviruses specifically, in addition to SARS-CoV-2, being male is a risk factor for more severe disease following infection with both SARS-CoV and middle-east respiratory syndrome virus (MERS) (30–33).

Sex differences in disease outcomes are not limited to infectious diseases but are also observed in outcomes of autoimmune diseases and cancers, for example. While males have a higher incidence of non-reproductive cancer and are generally more susceptible to severe outcomes from a broad variety of pathogens, including bacterial, parasitic, fungal and viral infections (10, 11, 34), females are more likely suffer from autoimmune disorders, including systemic lupus erythematosus and multiple sclerosis (35, 36). Inflammatory diseases, including those of the skin, respiratory, and gastrointestinal tract, also are more prevalent among adult females than males (37–43). Sex as a biological variable not only affects outcomes of diverse diseases but also impacts drug and vaccine efficacy (44). The latter being especially important in the context of the ongoing COVID-19 pandemic and world-wide SARS-CoV-2 immunization roll out (45–48).

## The Differences Between Biological Sex and Gender

**Figure 1:**
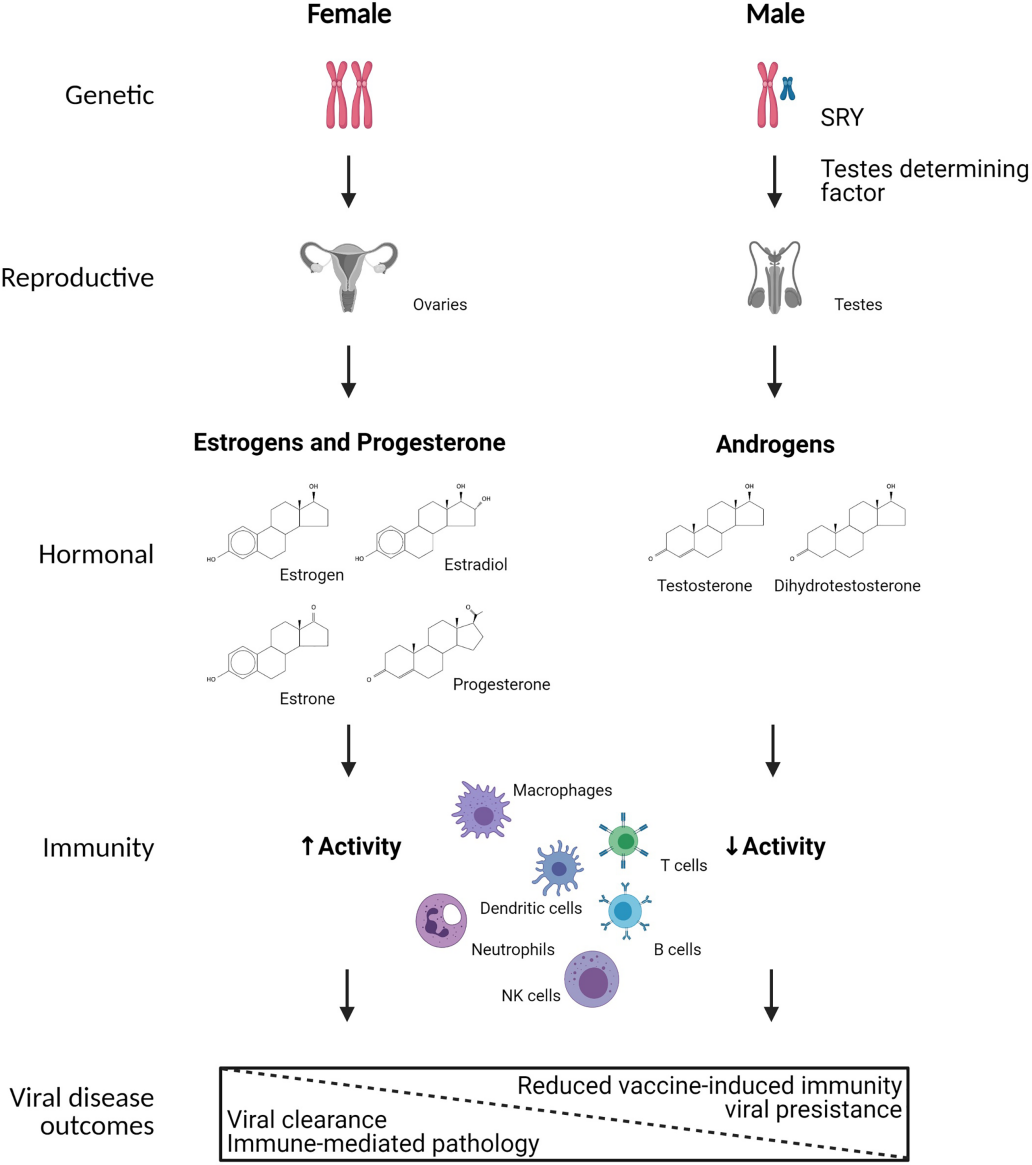
Mechanistic causes of sex differences in immunity to viral infection. Biological sex is defined by sex chromosome complement (i.e., XX or XY) in a majority of individuals, which results in sex differential development of gonadal tissues *in utero*, with development of ovaries in XX individuals and testes in XY individuals. The development of testes in XY individuals is primarily mediated by the expression of *SRY* on the Y chromosome, which encodes for testes determining factor. The ovaries and testes secrete differential concentrations of sex steroids, including estrogens, progesterone, and androgens. Numerous immune cells, including but not limited to macrophages, dendritic cells, neutrophils, NK cells, T cells, and B cells express cytoplasmic receptors for sex steroids, which can transcriptionally regulate gene expression, signal transduction, and responses of immune cells following viral infection. As a result, biological females tend to have greater immune system activation resulting in faster clearance of viruses, but also increased probability of developing immune-mediated pathology. In biological males, reduced immune system activation results in slower clearance of viruses, and in some cases viral persistence as well as reduced vaccine-induced immunity. Created using Biorender.

The terms ‘sex’ and ‘gender’ are often used interchangeably and, therefore, incorrectly. Sex refers to biological features that differ between males and females as a result of sex chromosome complement, the development of reproductive tissues, and concentration of sex steroids. Sex differences are often analyzed as a binary variable (i.e., male/XY *vs.* female/XX), but intersex individuals (i.e., individuals born with reproductive characteristics of both males and females) as well as Turner syndrome (i.e., XO individuals) and Kleinfelder’s syndrome (i.e., XXY individuals) patients provide evidence that sex occurs on a continuum, which deserves greater empirical consideration in the context of infectious diseases. Sex differences can affect the control and clearance of viruses which is primarily mediated by the immune responses initiated and the pathology that may occur following infection, which will be the focus of this review (**Figure 1**). Although not the focus of this review, anatomical sex differences in the genital tract are associated with increased transmission of certain pathogens, including sexually transmitted viruses, in females compared with males (38, 49).

Gender differences are reflected in the social-cultural construct of being a man, woman, or transgender (27). Gender differences reflect behaviour, cultural, and social factors that in the context of infectious diseases might impact exposures, access, and decision making about care or treatments. In a majority of individuals, biological sex (male or female) matches the subject’s gender (man or woman), with the biomedical implications of being transgender not thoroughly evaluated in the context of infectious diseases, including COVID-19. Other than consideration of whether transgender individuals trust of the medical establishment in the context of SARS-CoV-2 testing, COVID-19 treatment, and receipt of SARS-CoV-2 vaccines (50) as well as how receipt of gender-confirming hormone treatments and surgical procedures have been affected by the pandemic (51), there have been no reports comparing cis and transgender individuals in terms of infection rates or outcomes of either infection or vaccination against SARS-CoV-2. There also has been little to no COVID-19 tracking of data from transgender individuals at either country to state levels (9), which is a missed opportunity. There is, however, a call to include transgender individuals in studies evaluating the impact of sex steroids on COVID-19 outcomes (52).

Binary gender differences have been primarily characterized in the behaviors, occupations, and attitudes that impact risk of exposure to viruses, care-seeking behaviors, as well as access to care and reporting (11, 34). Importantly, cultural differences across the world may result in inconsistent or even contradictory observations about gender-specific differences in viral infections (53). Additionally, because sex and gender intersect, it is difficult to ascribe differential outcomes from viral infections in humans to either sex or gender. This is where animal models and primary cell culture systems can be integral for interpretations related to biological sex (54). Sex and gender further intersect with other demographic variables and comorbidities to influence viral infection outcomes. Obesity, for example, is more prevalent in females compared to males and is often linked to impaired immunity (55, 56). Male-female differences in the outcome of viral infections are also significantly affected by age as a result of genetic modifications as well as changing sex steroid concentrations throughout different stages of life (57). Females generally live longer than males, with increased frailty at older ages in females compared to males (58, 59). A longer life-span in females provides a natural bias in infection rates in this age group. Despite age-related changes in sex steroids, post-menopausal females remain immune-privileged even after the decline in sex steroid hormone concentration, suggesting genetic mechanisms are involved (58). Although the decline of sex steroids with age is more pronounced in females than males, the age-related decline of functional immunity paralleling this is slower in females compared to males (57, 60). An intersectional analysis provides a deeper understanding of the causes of differences between males and females in viral infection outcomes (61).

## Immunological Differences Between the Sexes

Several studies have shown that innate and adaptive immune responses are generally greater in females than males across diverse species (22, 34, 62, 63). The activity of innate immune cells, such as macrophages and dendritic cells (DCs), as well as the overall inflammatory response is generally greater in females than males, particularly during reproductive ages (62, 64). In humans, cytokine production following *ex vivo* stimulation of monocytes with lipopolysaccharide (LPS) is greater in cells from males than females, with evidence that hormone-based contraceptive use in females further reduces the production of cytokines, including IFN*γ* and TNFα (65). In response to SARS-CoV infection, male mice experience more severe outcomes within one week of infection, which corresponds with a more dramatic infiltration of inflammatory monocytes and neutrophils into the lungs of males as compared with females (66). Depletion of inflammatory monocytes in males resulted in SARS-CoV outcomes that are similar to females (66). Testosterone dampens infiltration of inflammatory monocytes and pulmonary inflammation during IAV infection (67). Macrophages from males are also more susceptible to infection with HIV-1 than are macrophages from females, which is at least partially caused by reduced virus restriction by SAMHD1 (i.e., SAM and HD domain containing deoxynucleoside triphosphate triphosphohydrolase 1) in cells from male donors (68). Plasmacytoid dendritic cells (pDCs) isolated from human females show greater expression of the X-linked gene, *TLR7*, and type 1 IFNs than pDCs from males, which is mediated by *TLR7* escape from X chromosome inactivation (69). Sex-based differences are reported in innate immune responses of pDCs to HIV-1-encoded TLR7 ligands, in which pDCs from HIV-1 infected women produce significantly more IFNα and show greater expression of interferon stimulated genes (ISGs), including interferon regulatory factor 5 (*IRF5*) than pDCs from HIV-1 infected men following *ex vivo* stimulation (70–72)

Females also have greater CD3^+^ and CD4^+^ T-cell counts as well a higher CD4^+^/CD8^+^ ratio compared to males, whereas frequencies of CD8^+^ T cells and NK cells are greater in males (73–78). Activity of both CD4^+^ and CD8^+^ T cells following stimulation is often greater in females than males (63, 72, 79). Additionally, antigen-presenting cells (APC) in female mice are reported to be more efficient in antigen presentation compared to APCs from male mice (80). Adult females present an immune response that is biased towards a more pro-inflammatory T_H_1 cytokine milieu while adult males show a predominant T_H_2 phenotype with increased frequencies of regulatory T cells (T_reg_) (22, 73, 81, 82). Adult females also have higher B-cell frequencies compared to males (74, 78). Basal levels and antibody response to viruses and vaccination also are greater in females than males (83–86). Because the pathogenesis of viruses is affected by both the hosts immune response and the virus, greater immunity among females can both be a cause and a consequence of viral disease (11). Specifically, the immune responses necessary to control virus replication, if excessive can cause immune-mediated pathology and tissue damage. While control of virus replication is often superior in females (e.g., HBV, HCV, and HIV), females also may experience more severe immune-mediated pathology (e.g., IAV and HIV) (11). A notable exception is SARS-CoV-2, in which males shed virus for a longer duration of time (87, 88) and present with greater inflammation (89) than females.

During SARS-CoV-2 infection, it has been reported that males present higher pro-inflammatory cytokine expression, including IL-6, than females (90, 91). While COVID-19 is known to cause lymphopenia in both sexes, T cell frequencies and activation remains greater in females than males during SARS-CoV-2 infection, even at older ages (91, 92). In contrast, male sex is associated with greater anti SARS-CoV-2 antibody production in convalescent patients (91, 93, 94) as well as fewer breakthrough SARS-CoV-2 infections following vaccination (95). Females have greater antibody responses to the mRNA SARS-Cov-2 vaccines than males (96).

## Sex Steroids Directly Affect Immunity to Viruses

Sex differences in immunity are often most pronounced in aged matched males and pre-menopausal females, with age-associated reductions in sex steroid concentrations paralleling changes in functional immunity (57, 58). Multiple mechanisms have been described for how sex steroids affect immune function directly and differentially in females and males. Sex steroids have been shown to directly affect gene expression on an epigenetic level (97, 98). Even viruses can directly interact with sex steroids; for example, HCV contains a progesterone response element suggesting that this virus can be regulated by the progesterone receptor (99).

Sex steroids affect the functioning of immune cells by binding to cytoplasmic receptors and interacting with nuclear hormone response elements (HRE) (100). Many genes involved in antiviral immunity, like *IFNG* and *IRF5*, possess estrogen response elements (63, 101, 102), and sex steroid mediated activation of HREs can directly result in increased cytokine and chemokine production (100). Expression of immune-regulatory miRNAs also can be under hormonal control (103). Estrogen receptors (ER) are differentially expressed among different immune cell subsets, with higher expression of ERα reported in T cells and high expression of ERβ found in B cells (102). In addition to classical nuclear signaling, non-classical ER signaling can affect many immune-related pathways, including through transcriptional regulation of NF-κB (104). Estrogens can have dose-dependent and bipotential effects on many immune cells (105–108). For example, estradiol (E2) affects the cytotoxicity of NK cells, activation and differentiation of monocytes, macrophages and dendritic cells, in a dose-dependent manner (109–112). Generally, low (non-pregnant) concentrations of E2 are thought to promote a T_H_1-based immune response, whereas high (pregnancy) E2 concentration, can drive the immune response more towards T_H_2 immunity (107). This is further supported by the fact that E2 at pregnancy doses enhances T_Reg_ proliferation (113). E2 induces somatic hypermutation and antibody class switching, with humoral responses to viral infection being greater in the presence as opposed to the absence of E2 (108, 114, 115).

Progesterone (P4) typically skews the immune profile of females from T_H_1 to either T_H_2 or T_reg_ immunity (116, 117). Progesterone receptors are found on many different immune cells, including T-cells, NK-cells, macrophages, and DCs (118). Progesterone reduces macrophage activation and production of pro-inflammatory cytokines in rodents and can antagonize TLR-signaling and signaling pathways involving NF-κB (119). Progesterone also dampens inflammation and promotes amphiregulin and Th17-mediated tissue repair after viral infection at mucosal sites, including the respiratory tract (120). As a result, P4-based therapies and contraceptives have been reported to reduce susceptibility to IAV infection, at last in mice (120, 121).

Androgens, in particular testosterone (T), are generally anti-inflammatory. Testosterone can decrease NK cell, neutrophil, and macrophage activity *in vitro* resulting in reduced production of pro-inflammatory cytokines, such as TNF-α and reactive oxygen species (e.g., iNOS and NO) (122–124). Testosterone can also increase the production of anti-inflammatory cytokines, such as IL-10 and TGF-β *via* androgen receptor signaling (124, 125). Production of anti-inflammatory cytokines (e.g., IL-10) after TLR9 activation, which is the pattern recognition receptor used for detection of DNA viruses, is greater in males and this effect positively correlates with androgen concentrations (126). Antibody response to vaccination are repeatedly described to be lower in males compared to females (83), with a T-sensitive gene cluster identified to correlate with lower vaccine-induced antibody responses in males (85).

The immunomodulatory function of T is most convincing in experimental studies in which T is removed and replaced exogenously. In the context of infection with IAV, T dampens pulmonary inflammation, including frequencies of inflammatory monocytes, eosinophils, and virus-specific CD8+ T cells after virus has been cleared (67). This effect is reversed by gonadectomy (i.e., surgical removal of the testes) or co-treatment of an androgen receptor antagonist in combination with T. In the context of vaccination, gonadectomy increases whereas treatment of gonadectomized male mice with T reduces vaccine-induced antibody responses (115). Aging is associated with reduced concentrations of T in male rodents and humans (115). Treatment of aged male mice with T improves the outcome of infection of IAV primarily by dampening inflammation as opposed to altering antiviral immunity and virus replication (127). Taken together there is growing evidence that immunity to viruses and vaccines is impacted by the concentrations of sex steroid hormones and signaling through their respective receptors, which impacts the outcome of viral infections differently for males and females over the life course, and in females with pregnancy.

## Genetic Factors Contribute to Sex Differences in Immunity to Viruses

Multiple studies highlight a significant role of sex chromosome complement on overall immunity (11, 128, 129). Many genes with immunomodulatory function are encoded on the X chromosome (130). The XX complement provides females with two copies of X chromosomes, one derived from the father and one derived from the mother. To compensate for gene dosage effects, one X chromosome is randomly inactivated in every single cell of a female. As this happens randomly, females show an X-chromosome mosaicism that provides many advantages compared to the XY complement. Disadvantageous mutations in one X-chromosome possibly affecting immune function will only affect half of the cells in a female, but necessarily all cells in a male. Furthermore, the XX chromosome complement provides females with additional allelic diversity that was hypothesized to be another advantage when facing new immune challenges (131–133). Genetic disorders like Klinefelter syndrome, resulting in males having an additional X chromosome (XXY) result in immune responses that are more similar to the typical female than male phenotype (134). Additionally, Turner’s syndrome, resulting in females with only one X chromosome (X0), is associated with lower lymphocyte counts and reduced antibody production in females (135). Sex chromosome complement is associated with multiple sex differences in diverse organs, even before gonadal development (129).

The X chromosomes encode for several immune-related genes such as *TLR7*, *IRAK1*, *FOXP3* as well as many miRNAs that are important for immune system gene regulation (136). Certain regions of the X-chromosome may escape inactivation with a direct effect on dosage compensation of X-linked immune genes (136, 137). TLR7, which is important for sensing RNA-viruses, is encoded on the X-chromosome and *TLR7* expression levels as well as immune sensing of RNA antigens, including virus vaccine and self-antigens are stronger in females (137, 138). Studies in the context of systemic lupus erythematosus show that substantial fractions of immune cells, including primary B lymphocytes, monocytes, and DCs express *TLR7* from both X-chromosomes, resulting in higher TLR7-driven functional responses in these cells (139, 140). Furthermore, TLR7 is an important mediator of B cell maturation and therefore antibody production (141). Greater expression of *TLR7* in B cells from females compared with males consequently is associated to greater antiviral antibody responses following receipt of the influenza vaccine in mice (142). In contrast, deleterious mutations in *TLR7*, which are only observed among males, can be associated with increased susceptibility to viral infections, including with SARS-CoV-2 (143). In addition to genes, a disproportionally high number of miRNAs is encoded on the X-chromosome (i.e., 10% of all miRNAs encoded in the human genome). In contrast, the Y-chromosome only encodes for two known miRNAs (144–146). There is evidence supporting that X-linked miRNAs are indeed a significant contributor to sex-differences in immunity (144).

Only biological males can be affected by Y chromosome (ChrY) polymorphisms, which can result in aberrated expression of immune-related genes (147, 148). This has primarily been studied in the context of autoimmunity, but also has implications on immunity to viral infections, including coxsackievirus and IAV (149). Strains of ChrY consomic mice have been used to show that variation in ChrY affects survival of male mice after IAV infection, which is associated with increased frequencies and activation of IL-17-producing γδ T cells that are linked to acute lung injury during IAV infection (150, 151). Polymorphisms in ChrY can affect global gene expression in immune cells through epigenetically mechanisms (149). The effect of ChrY polymorphisms on IAV pathogenesis are independent of sex steroids, underpinning the mechanistical features of genetic effects on sex-differences in immunity (152). Another important mechanism associated to the XY complement is loss of the Y chromosomes (LOY) primarily in leukocytes during aging, leading to widespread dysregulation of autosomal genes among leukocytes in men with possible implications on immunity (153–155). Taken together, sex chromosome complement, activity of regulatory elements on sex chromosomes, and the expression of sex chromosomal genes contribute to sex differences in immunity to viral infections.

## Sex Differences Immunity Begin *In Utero*

Sex differential mechanisms that are likely independent of sex steroids are also found in peri- and postnatal immunity and epigenetic imprinting during *in utero* development. There is increasing evidence that adverse prenatal conditions result in fetal epigenetic imprinting with long-term consequences on sex-specific immune system development. For example, prenatal micronutrition as well as maternal vitamin supplementation can have sex-differential effects on immunity in offspring, mostly on an epigenetic level (156, 157). Neurodevelopmental defects and neuroimmunological impairments observed in offspring born to mothers who experienced gestational stress have been shown to be sex-specific (158–160). Some studies suggest that female fetuses are more resistant to intra-uterine stress (161, 162). Postnatally, the protective effect of breast feeding seems to be stronger in female neonates compared to males, resulting in increased protection against respiratory infections in female neonates (163). Sex steroid concentrations do not differ between the sexes in infancy, except during the first three months of life, when male neonates express increased testosterone concentrations, followed by a gradual decline (164). Although most studies on maternal immune activation and *in utero* priming of the offspring are focused on neurodevelopment, there is increasing evidence from animal models that prenatal stress also affects the offspring’s immune-development in a sex-specific manner (165–167). Maternal immune activation in mice can disrupt the offspring’s immunological homeostasis with increased Th1 immunity in male offspring, particularly (165). Offspring born to dams that receive immunological stimulation (e.g., injection with lipopolysaccharide) also present with more activated, pro-inflammatory macrophages through adulthood (166); these studies, however, neglect consideration of the sex of the offspring.

The long-term effects of SARS-CoV-2 infection on offspring exposed to infection during pregnancy are currently not know. While most studies do not report vertical transmission of SARS-CoV-2, other mechanisms such as maternal and fetal hypoxia, disruption of placental integrity, or maternal immune activation and trans-placental transfer of pro-inflammatory cytokines must be assessed to understand possible implications on the offspring’s development and underlying sex-specific effects (168). Transfer of antibodies across the placenta might be an important contributor to sex-specific pathogenesis during SARS-CoV-2. While it has been shown that the neonatal Fc-receptor is crucial for antibody-transfer across the placenta, the placental interferon response affects Fc-receptor expression (169). Infection with SARS-CoV-2 during pregnancy results in sex differences in placental expression of interferon-stimulated genes and the Fc-receptor, with an upregulation in male fetuses, resulting in male-biased impaired transplacental antibody-transfer (169). Another study involving over 88.000 infants born in Sweden during the COVID-19 pandemic found a significant association between maternal SARS-CoV-2 infection and the absolute risk of respiratory and other neonatal disorders, but no increase in neonatal mortality. Unfortunately, neonatal outcomes were not sex disaggregated (170). Whereas several studies report that neonates born to SARS-CoV-2 positive women are more likely to need neonatal ICU admission, these data are generally not stratified for the offspring’s sex (171).

## Conclusions and Future Directions

There are sex differences in immunity to viruses that result in differential outcomes and pathogenesis of viral infections. Future studies should not only evaluate how sex differences in immunity alter the pathogenesis of viral infection and responses to vaccines, but consider the other pathways that may be differentially regulated between the sexes. For example, in the context of COVID-19, females show greater expression of type I IFN signaling and other innate immune responses and T cell-associated genes while cells from males exhibit greater expression of inflammatory genes (172). Sex differences in immunity are directly affected by sex chromosome complement through the differential activity of X-linked genes as well as ChrY gene polymorphisms that are regulated by escape from X inactivation and epigenetic mechanisms, respectively. The concentrations of sex steroids change over the life course, and directly affect immunity to viruses through the sex differential expression of sex steroid receptors in immune cells. Knowledge of sex differences in immunity to viruses and vaccines should inform greater consideration of sex disaggregation of data rather than statistical controlling for sex in clinical studies (173). Reporting of the sex of animals and primary cells as well as comparing immunological responses between the sexes in preclinical research is necessary (174). The COVID-19 pandemic has raised awareness about the significance of sex as a biological variable and how sex intersects with other variables, including age and race (45, 89, 91, 175). Intersectional approaches are going to be necessary to better understanding outcomes of viral infections as well as treatments for infection, with knowledge that both will likely differ between the sexes.

## Author Contributions

HJ outlined and wrote the first draft of the manuscript. SK edited and finalized the manuscript. SK conceived of the figure and HJ produced the figure. All authors contributed to the article and approved the submitted version.

## Funding

The writing of this review was made possible through funds from the NIH/NIA funded Johns Hopkins Specialized Center of Research Excellence (SCORE) in sex and age differences in immunity to influenza (SADII; U54AG062333).The funders had no role in the writing or content of this review.

## Conflict of Interest

The authors declare that the research was conducted in the absence of any commercial or financial relationships that could be construed as a potential conflict of interest.

## Publisher’s Note

All claims expressed in this article are solely those of the authors and do not necessarily represent those of their affiliated organizations, or those of the publisher, the editors and the reviewers. Any product that may be evaluated in this article, or claim that may be made by its manufacturer, is not guaranteed or endorsed by the publisher.
